# Nutritional interventions for adolescents using information and communication technologies (ICTs): A systematic review

**DOI:** 10.1371/journal.pone.0184509

**Published:** 2017-09-29

**Authors:** Giselle Rhaisa do Amaral e Melo, Fernanda de Carvalho Silva Vargas, Carolina Martins dos Santos Chagas, Natacha Toral

**Affiliations:** Department of Nutrition, University of Brasilia, Brasilia, Distrito Federal, Brazil; RTI International, UNITED STATES

## Abstract

A cost-effective and interactive way of promoting healthy nutrition behaviors among adolescents is using information and communication technologies (ICTs). We systematically reviewed studies to identify technologies and their main characteristics used for nutritional interventions for adolescents, as well as to evaluate their quality and effectiveness. Our full protocol is available on the PROSPERO website (#CRD42016035882). A search was conducted across five databases for articles describing nutritional interventions that used ICTs designed mainly for healthy adolescents. Randomized controlled trials, quasi-experimental and observational studies, and full and original papers, all of them published from 2005 to 2015, were included. Study quality was assessed by the Effective Public Health Practice Project Quality Assessment Tool. Our search yielded 559 titles and abstracts. Eleven studies met the inclusion criteria. Participants were recruited mostly from schools. Study follow-up ranged from two weeks to two years. Four interventions were based on the Social Cognitive Theory. Interventional strategies included computer games, programs, text messages, and interactive CD-ROMs. Nine studies used computer-mediated ICTs. Five studies focused on multiple behaviors simultaneously. Participants were exposed to interventions only once, daily, weekly, or according to a pre-determined number of lessons. Five studies had significant outcomes. Our quality assessment revealed three studies to be weak due to non-representativeness of their samples and usage of non-validated questionnaires. Besides the heterogeneity and poor quality of the analyzed studies, it can be suggested that long-term interventions for adolescents that make use of frequent exposure to technological resources, and that have a theoretical component aimed at a single health behavior change, tend to be more successful.

## 1. Introduction

Adolescence is considered a nutritional risk period marked by deep psychological, physiological, and social changes. In this age group, nutritional literature shows a prevalence of inadequate dietetic habits, such as high intake of processed or sugary foods, long gaps between meals, and low consumption of fruits and vegetables. The long-term effects of these eating patterns can result in overweight, as well as micronutrient deficiencies and chronic diseases that if left untreated can be dragged into adulthood [1].

Nutrition interventions are a cost-effective way to promote health behaviors and reduce obesity and chronic diseases among teens [1]. Use of the internet and technological resources is growing, especially among adolescents. As reported by the Pew Research Center in 2015, 92% of the American adolescents aged thirteen to seventeen indicated going online at least once a day. Furthermore, 88% and 87% mentioned having daily access to a mobile phone or to a desktop, laptop or computer, respectively [2].

A number of nutritional interventions, including some related to health promotion, have been delivered using information and communication technologies (ICTs), such as e-mails, websites, computer programs, smartphones, text messages, or games [3]. The use of web-based resources in the healthcare scene has given rise to a more innovative and interactive way of promoting behavior change and ultimately improving health programs [4]. Recently, Salam et al. highlighted the use of ICTs as an ideal platform for health interventions for teenagers, as it is considered to be “adolescent-friendly”. This approach could be useful even for disadvantaged adolescents or for those turned off by traditional health education methods [5].

Consequently, this systematic review identified the different technologies, as well as their main characteristics, that have been used for nutritional interventions directed at adolescents. A secondary objective was to evaluate the quality and effectiveness of these studies.

## 2. Methods

Our full protocol is available on the PROSPERO Website (#CRD42016035882). The intervention followed the PRISMA (Preferred Reporting Items for Systematic Reviews and Meta-Analyses) guidelines [6].

### 2.1. Data sources and search strategy

**Fig 1:**
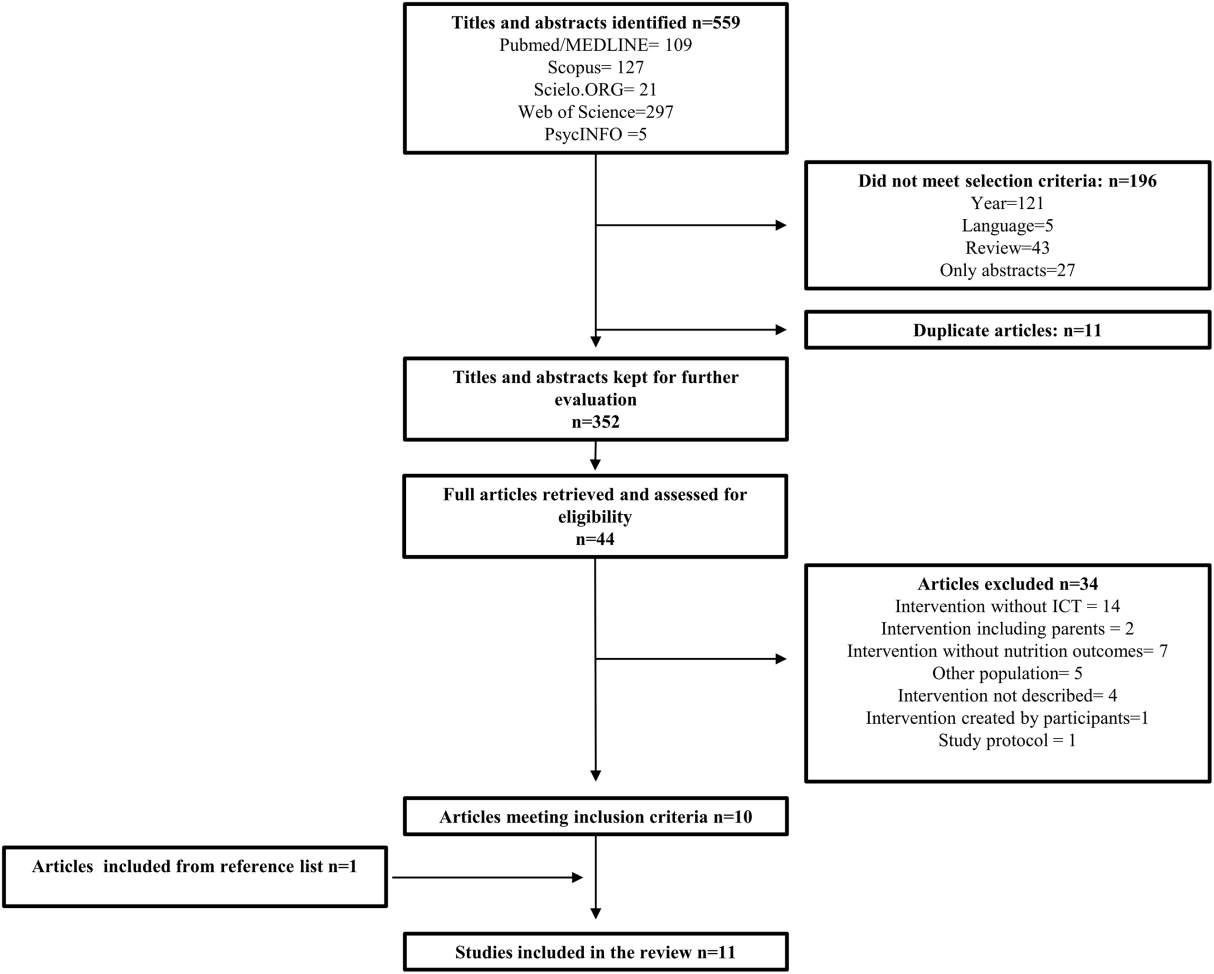
Search strategy for PubMed.

In order to find studies with valuable information about nutrition education interventions for adolescents that employed ICTs, five databases were searched: PubMed/MEDLINE, Scielo.ORG, Web of Science, PsycINFO, and Scopus. Several MeSH terms were applied to represent ICT (i.e., mobile health, cell phones, text message), nutrition (i.e., food consumption, eating patterns, dietary behavior), intervention (i.e., intervention study, training programs, health education), and adolescents (i.e., adolescence, teen, youth). Our full electronic search strategy for PubMed can be found in Fig 1. We reviewed articles in English, Portuguese and Spanish.

### 2.2. Selection criteria

Only randomized controlled trials, quasi-experimental and observational studies, full papers, and original articles, all of them published between January 2005 and January 2016, were considered for this review. The MeSH terms needed to be identifiable in the title or abstract. Description of the technology and/or intervention had to be available in the full paper. Participants were required to be healthy, but not necessarily to have a normal weight. Studies that included children older than eight or young adults could still be selected as long as they focused on adolescents between the ages of ten and nineteen.

### 2.3. Selection process

The list of titles and abstracts were downloaded and organized via the program *Mendeley*. Duplicates were removed and the remaining studies were sorted for eligibility by two reviewers. The full articles selected were then retrieved. Disagreements were resolved through discussion with an expert. Hand searches from reference lists of all included articles were performed. Both reviewers and the expert are registered dietitians with a background in this research area.

### 2.4. Assessment on quality and risk of bias

Study quality and risk of bias were assessed by the reviewers on study design, target population, confounders, data collection methods, dropouts, intervention integrity, and final analyses using the Effective Public Health Practice Project Quality Assessment Tool (EPHPP). Based on their final score, articles were classified as weak, moderate or strong. Those considered weak were not removed, but the risk of bias of their evidence was highlighted. Again, an expert adjudicated unresolved discrepancies.

### 2.5. Data collection process and synthesis

Our reviewers developed a data collection form based on the guidance from the Centre for Reviews and Dissemination on undertaking reviews in healthcare [7]. Independently, they extracted the data on type of publication, country, financial sources, main purpose, study design, inclusion/exclusion criteria, recruitment procedures, unit of allocation, participant characteristics (age, gender, ethnicity, social economic status or education, weight status, and comorbidities), intervention characteristics (type, frequency or duration of exposure, and theoretical basis), and outcomes (follow-up, dropout rate, type of analysis, and main and additional outcomes). Due to a lack of homogeneity within the included studies, a meta-analysis could not be performed. Data was then synthesized into a summary table.

## 3. Results

**Fig 2:**
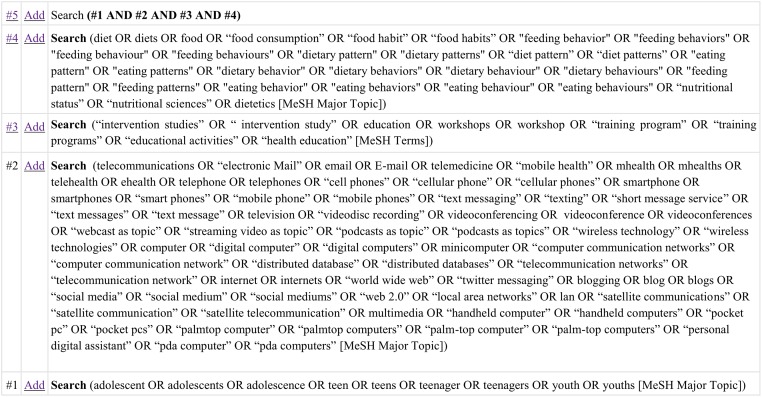
Retrieval and inclusion/exclusion process for articles used in review.

Our search yielded 559 titles and abstracts. Once they were screened, forty-four studies underwent further analyses. Eleven studies fully met the inclusion criteria, including one study that was found through hand searches of the reference lists. Fig 2 describes in detail the selection process of the included studies, and their main characteristics are listed in Table 1. Our quality assessment revealed three strong studies [8–10], five moderate ones [11–15], and three which were rated as weak [16–18]. These latter ones had a negative EPHPP evaluation due to a non-representativeness of their samples and use of non-validated questionnaires.

**Table 1:** Main characteristics of included studies.

*Study*	*Purpose of the study*	*Study design*	*Participants’ characteristics*	*ICT in Intervention*, *control and Theoretical Basis (TB)*	*Duration of exposure*, *follow-up and frequency*	*Main results*
Ezendam et al., 2012 [8]	To prevent weight gain in girls by improving dietary behaviors and physical activity.	Cluster randomized controlled trial	**Sample**: 883 **Age**: 12–13 **Gender**: Boys and girls **Country**: The Netherlands	**Intervention**: a web-based computer programme-tailored intervention **Control**: No-intervention **TB**: Theory of Planned Behavior	**Duration**: 10 weeks **Follow-up**: baseline, after 4 months and 2 years **Frequency**: 8 lessons (15min each)	Higher vegetables intake and lower snack and sugar-sweetened beverages consumption in the intervention group. Higher intake of fruit was found only on adolescents with a pre low intake in the intervention group.
Haerens et al., 2007 [9]	To evaluate the acceptability, feasibility and effectiveness of a computer-tailored education program created to reduce fat intake of adolescents.	Randomized controlled trial	**Sample**: 333 **Age**: 12–14 **Gender**: Boys and girls **Country**: Belgium	**Intervention**: Computer-tailored dietary fat intake intervention, provided as an interactive CD-ROM. **Control**: No-intervention **TB**: Social Cognitive theory, Theory of Planned behavior, and Transtheoretical model	**Duration**: 50 minutes **Follow-up**: baseline, after 3 months **Frequency**: 1 session	Decreased dietary fat intake in girls enrolled in technical-vocational schools; and in boys and girls who were in general education who reported reading intervention messages. No intervention effects for total sample.
Maes et al., 2011 [10]	To investigate the feasibility and impact of an Internet-based computer-tailored nutrition intervention.	Quasi-experimental design	**Sample**: 1298 **Age**: 12–17 **Gender**: Boys and girls **Country**: Austria, Belgium, Greece, Germany, Stockholm	**Intervention**: computer-tailored nutrition advice for improving dietary intake of target nutrients (fiber, vitamin C, calcium, iron and fat) **Control**: Generic standard advice in text format covering similar topics **TB**: Not informed	**Duration**: not informed **Follow-up**: baseline, after 1 and 3 months **Frequency**: 3 sessions during school hours	No significant changes in fat intake for the intervention group
Lubans et al., 2012 [11]	To evaluate the impact of a school-based obesity prevention program for adolescents.	Cluster randomized controlled trial	**Sample**: 357 **Age**: 12–14 **Gender**: Girls only **Country**: Australia	**Intervention**: multi-component school-based intervention program, including text messages, nutrition workshops, interactive seminars, handbooks, and sports sessions. **Control**: No-intervention **TB**: Social Cognitive Theory	**Duration**: 12 months **Follow-up**: baseline after 12 months **Frequency**: weekly or bi-weekly text messages	No significant changes in BMI and body fat percentage in the intervention.
Rees et al., 2010 [12]	To evaluate the effectiveness of a computer-generated tailored intervention to increase intakes of brown bread, wholegrain cereal, fruits, and vegetables.	Cluster randomized controlled trial	**Sample**: 823 **Age**: 12–16 **Gender**: Girls only **Country**: United Kingdom	**Intervention**: Computer-tailored intervention, based on individual’s self-reported intake of target foods. **Control**: Generic leaflet based on National Guidelines (not tailored) **TB**: Theory of Planned Behavior, and The Transtheoretical Model	**Duration**: not informed **Follow-up**: baseline, after 3 months **Frequency**: 1 session	The tailored intervention leaflet had a significant effect on whole bread intake, but there were no significant effects for other foods.
Sharma et al., 2015 [13]	To evaluate the feasibility, acceptability, and effects of a computer game on dietary behaviors, physical activity behaviors, and psychosocial factors.	Quasi-experimental design	**Sample**: 107 **Age**: 9–11 **Gender**: Boys and girls. **Country**: United States of America	**Intervention**: a game in which players must create an avatar and make it eat healthy and stay active; and complete a series of progressive gaming challenges. **Control**: No-intervention **TB**: Social Cognitive Theory and the Theory of Reasoned Action	**Duration**: 6 weeks **Follow-up**: baseline, after 6 weeks **Frequency**: 90 minutes per week	The intervention group had lower sugar consumption and improved nutrition and physical activity attitudes post intervention compared to the control group.
Thompson et al., 2009 [14]	To evaluate the effects of a Boy Scout program on fruit juice and low-fat vegetable consumption.	Cluster randomized controlled trial	**Sample**: 473 **Age**: 10–14 **Gender**: Boys only **Country**: United States of America	**Intervention**: a website intervention to increase fruit juice and low-fat vegetable consumption with online activities (knowledge games, web recipes, goal setting, problem solving) **Control**: Mirror-image intervention to increase physical activity **TB**: Social Cognitive Theory	**Duration**: 9 weeks **Follow-up**: after 9 weeks and 6 months **Frequency**: 55 minutes per week	Significant increases in fruit juice consumption, fruit juice home availability, and low-fat vegetable self-efficacy in the intervention group immediately following the intervention but were not maintained 6 months later.
Whittemore et al., 2012 [15]	To compare the effectiveness of two school-based internet obesity prevention programs.	Cluster randomized controlled trial	**Sample**: 604 **Age**: 14–16 **Gender**: Boys and girls **Country**: United States of America	**Intervention**: Two experimental groups: - A program including lessons (nutrition, physical activity, metabolism and portion control), self-monitoring, health coaching, and social networking. - The same above plus coping skills training (addition of 4 lessons on coping skills) **Control**: No-intervention **TB**: Social Learning Theory	**Duration**: not informed **Follow-up**: after 3 and 6 months **Frequency**: 8 to 12 lessons	Both groups significantly improved health behaviors including self-efficacy, healthy eating, fruit and vegetable intake, moderate and vigorous exercise, and stretching exercises; decreases in consumption of sugar-sweetened beverages and junk food, and decreased sedentary behavior.
Baños et al., 2012 [16]	To investigate the efficacy for improving nutritional information and evaluate acceptability and playability of an online game.	Quasi-experimental design	**Sample**: 228 **Age**: 10–13 **Gender**: Boys and girls **Country**: Spain	**Intervention**: ETIOBE mates, educational website including games **Control**: Paper-pencil intervention **TB**: Not informed	**Duration**: 2 weeks **Follow-up**: baseline after 2 weeks **Frequency**: “as much as they wanted”	Improved nutritional knowledge for both groups. Scores were greater in the intervention group.
Bech-Larsen & Grønhøj, 2013 [17]	To increase the consumption of fruits and vegetables of Danish adolescents as well as evaluate the SMS intervention in terms of participation, goal setting, and performance of adolescents.	Cluster Randomized controlled trial	**Sample**: 256 **Age**: 12 **Gender**: Boys and girls **Country**: Denmark	**Intervention**: SMS-based diary and feedback system plus nutrition education **Control**: Nutrition education only **TB**: Not informed	**Duration**: 4 weeks **Follow-up**: baseline, after 15 weeks **Frequency**: daily SMS messages	Increased frequency of fruits and vegetables consumption only for those with a low pre-intervention intake in the intervention group.
Yang et al., 2015. [18]	To improve intake of food groups and nutritional elements using technology based team game.	Quasi-experimental design	**Sample**: 87 **Age**: 15–16 **Gender**: Girls only **Country**: Taiwan	**Intervention**: Two experimental groups: - Use of a Diet Assessment System for self-monitoring and metacognitive strategies; - The Diet Assessment System was also used as an online team-based competitive game **Control**: Traditional lecture-based instruction plus motivational elements (video clips related to healthy eating) **TB**: Social-interdependence theory/ social learning	**Duration**: 8 weeks **Follow-up**: baseline, after 8 weeks **Frequency**: 50 minutes per week	Group 2 improved dietary behaviors of most food groups (dairy, meats, proteins, vegetables and fruits), macronutrients (calories and fiber), and micronutrients (calcium, vitamin C and B2). Improvements were greater in Group 2 compared to the other two groups.

FFQ, Food Frequency Questionnaire; BMI, Body Mass Index; ICTs: information and communication technologies.

### 3.1. Population

Participants were recruited from school settings [8–13, 15–18], with the exception of one study that recruited boy scout troops [14]. Participant age ranged from nine to seventeen. Four studies focused on twelve- to fourteen-year-olds [8–9, 11, 17], four included older participants (fifteen- to seventeen-year-olds) [10, 12–13, 18], and three encompassed younger ones [13–14, 16]. All studies included participants from developed countries, but five interventions targeted specific populations such as low-income groups, ethnic minorities, or minority females or males [11–14, 18].

### 3.2. Study design

Sample sizes ranged from 87 to 1298 participants, and three studies observed over 800 adolescents [8, 10, 12]. Of the eleven interventions, two were randomized controlled trials [11, 17], five were cluster randomized controlled trials [9–10, 13, 15, 18], and four used a quasi-experimental design [8, 12, 14, 16].

### 3.3. Follow-up and study duration

The majority of studies had one follow-up measurements after baseline assessment [9, 11–13, 16–18], but four had two [8, 10, 14–15]. Study duration (from beginning to last follow-up assessment) ranged from two to eight weeks [13, 16, 18], three to four months [9–10, 12, 17], six months [14–15], and one to two years [8, 11]. Three of the four studies which had a follow up after six months did not maintain the significant results obtained immediately post-intervention [8, 14–15].

### 3.4. Theoretical basis

Four interventions used the Social Cognitive Theory as a theoretical basis, either alone [11, 15] or in combination with the Theory of Reasoned Action [13] or the Theory of Planned Behavior [9]. Two studies based its interventions on principles from the Social Learning Theory, which supports the role of social and affective elements in behavior change and in the adoption of healthy behaviors [15, 18].

### 3.5. Intervention strategies and measured variables

Four interventions adopted computer games [13–14, 16, 18], four utilized computer programs that generated tailored feedback or advice [8, 10, 12, 15], two used text messages [11, 17] and one used an interactive CD-ROM [9]. Altogether, nine out of our eleven studies employed computer-mediated interventions, in the form of a program, website, game, or email tailored feedback [8–10, 12–16, 18]. Only two studies dealt with smartphones [11, 17], including one which focused exclusively on this type of technology [11]. Five of the eleven interventions investigated dietary intakes of multiple food groups and nutrients simultaneously [8, 10, 12–13, 15], two focused only on fruits and vegetables [14, 17], and the remainder evaluated either dietary fat intake alone [9], total calories per day [11], or nutritional knowledge [16]. Components such as physical activity [10–11, 13], psychological variables [12], and psychosocial factors [13–14] were also incorporated or evaluated in some interventions. Four of eleven interventions analyzed both healthy eating and physical activity behaviors [8, 11, 13, 15].

### 3.6. Intervention duration and frequency of exposure

Duration and frequency of exposure to intervention varied widely. In two studies participants were exposed to intervention only once [9, 12], whereas other programs had daily [17] or weekly activities [11, 13–14, 16, 18]. There were also studies that did not set a specific time of exposure to intervention, but rather established a number of lessons to be done within one to three months [8, 10, 15]. Detailed information on each intervention can be found in Table 1.

### 3.7. Main outcomes

Five interventions had positive effects on diet that were statistically different from their baseline measurements and/or comparison group [8, 13–15, 18]. Ezendam et al. found an increased intake of vegetables and a decrease in snack and sugar-sweetened beverages four months after their intervention, but these findings had regressed after two years [8]. A lower sugar consumption was also shown by Sharma et al. and Whittemore et al., whose interventions similarly resulted in a decrease in junk food intake associated with an increase in vegetable and fruit consumption [13, 15]. Thompson et al. found that boy scout troops underwent significant increases in fruit juice consumption and home availability immediately post-intervention, but these results were not maintained later [14]. Bech-Larsen & Gronhoj also found a significant increase in fruit and vegetable consumption, but only for students who had a low baseline intake of these food groups [17]. Ress et al. did not show statistically positive interventional effects in favor of fruits and vegetables, but did show an increase of brown bread servings in their experimental group [12]. Yang et al., who evaluated the effects of a team-based approach to interventions, showed significant improvements in the consumption of most food groups (dairy, meats, fruits, and vegetables), as well as an increase in the consumption of fiber, calcium, and vitamins C and B2, compared to baseline measurements and to a second experimental group who underwent a more individualized intervention rather than a group interaction [18]. Haerens et al. did not detect effects of their intervention in their sample as a whole, although a decrease in fat consumption was observed in girls from technical-vocational schools and in both girls and boys from general schools [9]. Maes et al. reported a gradual increase of fat intake in their control group; however, fat intake in their intervention group remained stable [10]. Baños et al. found a significant increase in nutritional knowledge for both their groups, although a higher score was observed across the intervention students [16].

## 4. Discussion

We systematically reviewed eleven studies, all of which used ICT-based interventions designed mainly for adolescents. This review is unique in that it presents studies that include all types of technology within the scope of the adoption of healthy eating habits, rather than focusing exclusively on weight status or obesity. Furthermore, the main objective of this review is to examine the trends in the ICTs used in interventions for teens in the last 10 years, and the description of their effectiveness is only a consequence, not our focus. This overview identifies current gaps in these trends and allows researchers to design innovative interventions so that the scientific community can better develop programs aimed at youths, allowing a wider range of technologies and their efficiency to be tested.

The majority of studies recruited individuals from schools. According to Hoelsher et al., this can be helpful in providing a continuous contact with the participants, in addition to rendering the research more cost-effective. Only one of the studies whose population were minorities failed to present at least one significant outcome in their intervention groups compared to their control groups. Ricci-Cabello et al. and Nierkens et al. concluded that educational programs aimed at minorities have the potential to be more effective; however, this fact can be easily influenced by the design, duration of intervention and follow up, and sample size of the study. Further research, including more homogeneous nutritional studies, needs to be explored [1, 19–20].

Both studies which exposed participants to their interventions only once or on a daily basis presented significant immediate results. The long-term effects of the interventions, however, were not maintained. It is clear that continuous interventions are needed for outcomes to be tracked into adulthood, but the specific amount of time cannot be answered by our study. These findings are consistent with Norman et al. and Shaya et al. [21, 22]

The predominant theoretical basis among the studies we reviewed was the Social Cognitive Theory. Although all the studies using this theoretical framework showed immediate post-intervention significant outcomes, it cannot be determined whether this phenomenon is a result of this particular theory or not. Health behavior change is complex and involves social, emotional, and cognitive determinants that ultimately influence how people adopt certain eating behaviors. These theories and models focus on understanding how these determinants influence health behaviors in order to guide future interventions. They tend to use different constructs and different theories are often used in combination. It is worth suggesting that the use of more than one health behavior theory or model can be beneficial in promoting healthy eating, because different elements are considered [21, 23].

Among the intervention strategies we analyzed, only one used a CD-ROM, showing that new types of ICTs, such as games, are emerging in the healthcare scene. In this systematic review, all interventions using games have shown to be effective; this can be attributed to the entertaining way in which these interventions promote educative learning, which appeals to younger populations [16]. The exclusively-smartphone-based study had positive outcomes, but our quality assessment rated is as weak, showing room for improvement in future researches. Successful interventions require identifying the types of technology most present in adolescents’ routines; doing so facilitates the availability of information, as these devices are already a part of the adolescent’s environment [24].

Since this systematic review found exclusively studies of high income countries, and only one of them targeted low income groups, it is hard to state conclusions about the use of ICT-based interventions in undeveloped countries. Guse et al. systematically reviewed the use of digital media to improve adolescent sexual health and found evidence to support the need of technological support and logistical help in countries with less access to technologies [25].

Most interventions reported being effective in promoting a variety of health benefits related to nutrition. Six interventions in this systematic review compared ICT-based to face-to-face programs and only a single study failed to present a superior outcome for the ICT-based intervention group. However, these studies have a short follow-up, and thus additional comparisons are necessary to prove ICT-based interventions are superior or equally effective as traditional programs for the maintenance of healthy eating behaviors in adolescents.

Regarding the advantages of ICT-based interventions, it is believed this type of delivery platform provide more personalized content and feedback for the participants in comparison to traditional approach. This feature is especially important for the promotion of healthy eating behaviors, since the participants need to incorporate the information obtained in their daily routine. Guse et al. and Lau at al found similar results in systematic reviews exploring ICT-based interventions for promoting sexual health and physical activity behavior, respectively [23, 25]. In addition, ICT-based interventions deliver the information in a variety of forms, such as videos and games, which seemed to be more attractive, draw attention and be easily remembered, which facilitate the communication with this age group [13, 23].

A review of school-based educational programs for teens for the promotion of physical activity and health eating behaviors concluded that the use of games can increase motivation, therefore promoting the acquisition of healthy eating habits [26]. Another benefit of ICT-based interventions is that they are not time-consuming. Most programs took less than an hour per week to be completed. Generic nutrition content, low motivation of participants associated with lack of time are commonly limitations of traditional intervention programs for adolescents that can be addressed with the use of technology [23, 26–28]. However, differently from face-to-face interventions, depending on the platform adopted in a ICT-based intervention, researchers cannot truly guarantee who is actually interacting with the program (i.e. a friend can play a game displayed in a smartphone instead of the study participant), although there are a number of strategies that can be used to address this issue. Per instance, a study included in this review allowed students to play only in computers located at schools [13].

It seems that studies targeting a single behavior, such as those focused on fruit and vegetable consumption, had better outcomes after their interventions. Although this evidence is questionable due to the weak or moderate quality of some studies as well as short duration of follow-up and small sample size, it is in accordance with Norman et al. [21]. Targeting healthy eating associated with physical activity seems to be have a greater impact in adolescents’ health, especially on weight, since several aspects of health are being taken into consideration. Half of the studies which combined those behaviors presented positive outcomes in both variables. Despite questionable evidence due to the low number of studies, a systematic review which determined the effectiveness of interventions that focus on improving diet and physical activity behaviors in school children concluded combined interventions, mostly, present a longer lasting effectiveness of the intervention [29].

This systematic review found a number of heterogeneous studies which makes it difficult to simply synthesize our gathered data. For better scientific insights, experimental studies should be conducted with only one type of ICT component, in a strong-evidence design. Based on Whiteley et al., a greater understanding on the impact of ICTs can only be achieved once future research address randomization, representativeness of the population, and sufficient length of duration and follow-up of the intervention [30]. It is also important to use standardized and validated measurement instruments to maximize comparability of results, especially in studies focusing on behavior change interventions among adolescents [5].

## 5. Conclusion

The advantages of using technology in nutritional education programs for adolescents have been evidenced. This method of delivering information has the benefit of being more interactive, and personalized, and holds greater traction among youths. As new types of ICTs are currently emerging, the results of intervention studies are basically preliminary. The heterogeneity of these studies makes it hard to state what type of intervention is more effective; however, we can suggest that long-term interventions with frequent exposure to technological resources that also have a theoretical component aimed at a single health behavior change can potentially improve nutrition behaviors.

## Supporting information

S1 Table EPHPP’s evaluation. (XLSX)

S2 Table Data extraction table. (XLSX)

S1 File PROSPERO protocol. (PDF)

S2 File PRISMA 2009 checklist. (DOC)
