# Supplementary material for: Nutritional interventions for adolescents using information and communication technologies (ICTs): A systematic review
Source: PLoS One. 2017 Sep 29;12(9):e0184509. doi: 10.1371/journal.pone.0184509 (PMC5621667; doi:10.1371/journal.pone.0184509)
Supplement: S1 File — (PDF) [file pone.0184509.s003.pdf]

University of York
Centre for Reviews and Dissemination

NHS
National Institute for Health Research

PROSPERO International prospective register of systematic reviews

Review title and timescale

1 Review title
Give the working title of the review. This must be in English. Ideally it should state succinctly the interventions or exposures being reviewed and the associated health or social problem being addressed in the review.
Nutritional interventions for adolescents using information and communication technologies (ICT): a systematic review

2 Original language title
For reviews in languages other than English, this field should be used to enter the title in the language of the review. This will be displayed together with the English language title.

3 Anticipated or actual start date
Give the date when the systematic review commenced, or is expected to commence.
01/12/2015

4 Anticipated completion date
Give the date by which the review is expected to be completed.
01/04/2016

5 Stage of review at time of this submission
Indicate the stage of progress of the review by ticking the relevant boxes. Reviews that have progressed beyond the point of completing data extraction at the time of initial registration are not eligible for inclusion in PROSPERO. This field should be updated when any amendments are made to a published record.

The review has not yet started ✖

Review stage	Started	Completed
Preliminary searches	No	Yes
Piloting of the study selection process	No	Yes
Formal screening of search results against eligibility criteria	No	Yes
Data extraction	No	No
Risk of bias (quality) assessment	Yes	No
Data analysis	No	No

Provide any other relevant information about the stage of the review here.

Review team details

6 Named contact
The named contact acts as the guarantor for the accuracy of the information presented in the register record.
Giselle Rhaisa do Amaral e Melo

7 Named contact email
Enter the electronic mail address of the named contact.
giselle-melo1502@hotmail.com

8 Named contact address
Enter the full postal address for the named contact.
Natacha Toral Secretaria de Nutrição, Faculdade da Saúde Campus Universitário Darcy Ribeiro, Universidade de Brasília Asa Norte, Brasília- DF, Brazil Cep: 70910-900

9 Named contact phone number
Enter the telephone number for the named contact, including international dialing code.

10 Organisational affiliation of the review
Full title of the organisational affiliations for this review, and website address if available. This field may be completed as 'None' if the review is not affiliated to any organisation.
Universidade de Brasília
Website address:
http://fs.unb.br

11 Review team members and their organisational affiliations
Give the title, first name and last name of all members of the team working directly on the review. Give the organisational affiliations of each member of the review team.

Title	First name	Last name	Affiliation
Miss	Giselle Rhaisa	do Amaral e Melo	Universidade de Brasília
Miss	Fernanda	de Carvalho Silva Vargas	Universidade de Brasília

Dr	Maria Natacha	Toral Bertolin	Universidade de Brasília
Ms	Carolina	Martins dos Santos Chagas	Universidade de Brasília

12 Funding sources/sponsors

Give details of the individuals, organizations, groups or other legal entities who take responsibility for initiating, managing, sponsoring and/or financing the review. Any unique identification numbers assigned to the review by the individuals or bodies listed should be included.

none

13 Conflicts of interest

List any conditions that could lead to actual or perceived undue influence on judgements concerning the main topic investigated in the review.

Are there any actual or potential conflicts of interest?

None known

14 Collaborators

Give the name, affiliation and role of any individuals or organisations who are working on the review but who are not listed as review team members.

Title	First name	Last name	Organisation details

Review methods

15 Review question(s)

State the question(s) to be addressed / review objectives. Please complete a separate box for each question.

What type of nutritional education interventions using digital technology are being applied for adolescents ?
what are their impact on nutrition behavior?

16 Searches

Give details of the sources to be searched, and any restrictions (e.g. language or publication period). The full search strategy is not required, but may be supplied as a link or attachment.

Eletronic searches up to January 2016 will be conducted using the following electronic bibliographic databases: Medline/PubMed, Scopus, Web of Science, PsycINFO, and SciELO.ORG. The initial search strategy will be designed for PubMed/Medline and adapted to other databases. After the study selection, we will search the reference list of included articles. It will be included studies that were written in Portuguese, English or Spanish and published between January 2005 and January 2016. Only full papers, original articles, are going to be included.

17 URL to search strategy

If you have one, give the link to your search strategy here. Alternatively you can e-mail this to PROSPERO and we will store and link to it.

18 Condition or domain being studied

Give a short description of the disease, condition or healthcare domain being studied. This could include health and wellbeing outcomes.

Nutrition. Healthy eating habits in adolescents.

19 Participants/population

Give summary criteria for the participants or populations being studied by the review. The preferred format includes details of both inclusion and exclusion criteria.

Healthy adolescents (10 to 19 years old). We will also include those studies which mainly focused on adolescents, but included children above 8 yo or young adults. Overweight will not be considered as a disease.

20 Intervention(s), exposure(s)

Give full and clear descriptions of the nature of the interventions or the exposures to be reviewed

We will include studies that promote valuable information about nutritional education interventions or programs related to any field of nutrition that used ICT in adolescents.

21 Comparator(s)/control

Where relevant, give details of the alternatives against which the main subject/topic of the review will be compared (e.g. another intervention or a non-exposed control group).

None or traditional nutrition education OR different technologies

22 Types of study to be included initially

Give details of the study designs to be included in the review. If there are no restrictions on the types of study design eligible for inclusion, this should be stated.

Randomized controlled trials, quasi-experimental, and observational studies will be considered

23 Context

Give summary details of the setting and other relevant characteristics which help define the inclusion or exclusion criteria.

24 Primary outcome(s)

Give the most important outcomes.

What are the main characteristics of the intervention (materials, frequency of exposure, variables measured, settings, etc)?

Give information on timing and effect measures, as appropriate.

25 Secondary outcomes

List any additional outcomes that will be addressed. If there are no secondary outcomes enter None.

Effectiveness of the interventions.

Give information on timing and effect measures, as appropriate.

26 Data extraction, (selection and coding)

Give the procedure for selecting studies for the review and extracting data, including the number of researchers involved and how discrepancies will be resolved. List the data to be extracted.

In phase 1, two reviewers(1R and 2R) will review independently the list of titles and abstracts according to the inclusion criteria. The full articles selected for phase 2 will be read independently by two reviewers (1R and 2R) to decide whether they meet the inclusion criteria. If there is disagreement in screening and eligibility phases, the expert (E) will be contacted to make the decision. Data will be extracted independently by two reviewers (1R and 2R) on the following characteristics: quality of the study, author and year of publication, sample, age of the sample, type of technology, description of the education intervention, length of the intervention, outcomes and conclusions. Disagreements will be resolved by the expert . Others papers can be added in the review via reference lists from original papers. Articles will be organized via the program Mendeley.

27 Risk of bias (quality) assessment

State whether and how risk of bias will be assessed, how the quality of individual studies will be assessed, and whether and how this will influence the planned synthesis.

Two reviewers (1R and 2R) will perform the quality assessment of the included studies. Disagreements will be revised by discussion, and the expert (E) will adjudicate unresolved discrepancies. The quality of the studies will be described in table and the Effective Public Health Practice Project (EPHPP) Quality Assessment Tool will be used.

28 Strategy for data synthesis

Give the planned general approach to be used, for example whether the data to be used will be aggregate or at the level of individual participants, and whether a quantitative or narrative (descriptive) synthesis is planned. Where appropriate a brief outline of analytic approach should be given.

The characteristics of interest of the studies will be described in table as informed in “Data collection process”. The interventions will be described in a narrative way. Data will be used aggregated.

29 Analysis of subgroups or subsets

Give any planned exploration of subgroups or subsets within the review. ‘None planned’ is a valid response if no subgroup analyses are planned.

none planned.

Review general information

30 Type of review

Select the type of review from the drop down list.

Intervention

31 Language

Select the language(s) in which the review is being written and will be made available, from the drop down list. Use the control key to select more than one language.

English

Will a summary/abstract be made available in English?

Yes

32 Country

Select the country in which the review is being carried out from the drop down list. For multi-national collaborations select all the countries involved. Use the control key to select more than one country.

Brazil

33 Other registration details

Give the name of any organisation where the systematic review title or protocol is registered together with any unique identification number assigned. If extracted data will be stored and made available through a repository such as the Systematic Review Data Repository (SRDR), details and a link should be included here.

34 Reference and/or URL for published protocol

Give the citation for the published protocol, if there is one.

Give the link to the published protocol, if there is one. This may be to an external site or to a protocol deposited with CRD in pdf format.

35 Dissemination plans

Give brief details of plans for communicating essential messages from the review to the appropriate audiences.

Do you intend to publish the review on completion?

Yes

36 Keywords

Give words or phrases that best describe the review. (One word per box, create a new box for each term)

eHealth

Adolescent
Health Education,
Nutrition

37 **Details of any existing review of the same topic by the same authors**
Give details of earlier versions of the systematic review if an update of an existing review is being registered, including full bibliographic reference if possible.

38 **Current review status**
Review status should be updated when the review is completed and when it is published.
Ongoing

39 **Any additional information**
Provide any further information the review team consider relevant to the registration of the review.
This review is being undertaken as a preliminary step for the development of an eHealth intervention for brazilian adolescents.

40 **Details of final report/publication(s)**
This field should be left empty until details of the completed review are available.
Give the full citation for the final report or publication of the systematic review.
Give the URL where available.
